# Towards a Highly Sensitive Piezoelectric Nano-Mass Detection—A Model-Based Concept Study

**DOI:** 10.3390/s21072533

**Published:** 2021-04-04

**Authors:** Jens Twiefel, Anatoly Glukhovkoy, Sascha de Wall, Marc Christopher Wurz, Merle Sehlmeyer, Moritz Hitzemann, Stefan Zimmermann

**Affiliations:** 1 Institute of Dynamics and Vibration Research, Leibniz Universität Hannover, An der Universität 1 Geb. 8142, 30823 Grabsen, Germany; 2 Institute of Micro Production Technology, Leibniz Universität Hannover, An der Universität 2, 30823 Grabsen, Germany; glukhovskoy@impt.uni-hannover.de (A.G.); dewall@impt.uni-hannover.de (S.d.W.); wurz@impt.uni-hannover.de (M.C.W.); 3 Institute of Electrical Engineering and Measurement Technology, Leibniz Universität Hannover, Appelstr. 9A, 30167 Hannover, Germany; sehlmeyer@geml.uni-hannover.de (M.S.); hitzemann@geml.uni-hannover.de (M.H.); zimmermann@geml.uni-hannover.de (S.Z.)

**Keywords:** piezoelectric sensors, nano-mass detection, inertial balance, resonance systems, nano/micro-electro-mechanical-system, N/MEMS, co-resonance

## Abstract

The detection of exceedingly small masses still presents a large challenge, and even though very high sensitivities have been archived, the fabrication of those setups is still difficult. In this paper, a novel approach for a co-resonant mass detector is theoretically presented, where simple fabrication is addressed in this early concept phase. To simplify the setup, longitudinal and bending vibrations were combined for the first time. The direct integration of an aluminum nitride (AlN) piezoelectric element for simultaneous excitation and sensing further simplified the setup. The feasibility of this concept is shown by a model-based approach, and the underlying parameter dependencies are presented with an equivalent model. To include the geometrical and material aspects, a finite element model that supports the concept as a very promising approach for future nano-mass detectors is established.

## 1. Introduction

The detection of weight is an important measurement task that has been performed for thousands of years. Over time, precision and repeatability has dramatically improved. However, the measurement of exceedingly small masses—of the order of the atomic mass unit Dalton—is still a great challenge, even today. An important method for the detection of the smallest masses is the use of resonance modes, wherein the resonance frequency is detuned by the mass to be detected. This method was first described in [1] in 1959 and has been since then cited more than 9000 times. At that time, an accuracy of 10^−10^ g was already archived with eigenfrequencies in the order of 10 MHz. Such an inertial balance utilizes the piezoelectric effect of the vibrating element, which is exited at a fundamental resonance via a closed loop control (e.g., [2]). This can be realized by either a phase-locked-loop or a self-oscillating circuitry. The frequency dependence of the resonance on the analyte mass is due to the ratio of analyte mass to the effective mass of the resonator; the highest sensitivity is obtained when both masses are similar in weight. Consequently, research in this field has focused on even smaller and lighter resonator structures while aiming for better mass resolutions. The smallest known resonators are carbon nanotubes, with flexural mode resonances in the GHz range that allow for yoctogram mass resolutions [3]. However, the constantly decreasing sizes bring two major challenges: first, the fabrication gets more complicated and therefore more expensive, making reproducibility difficult; second, the vibration measurement increases in complexity with decreasing structure size.

In recent years, many research groups have worked on improving inertial balances, as listed in Table 1. Various concepts of actuation and mass sensing have been evaluated including inductive, electrostatic, piezoelectric, and piezoresistive methods [4]. The maximum obtained resolution was 10^−24^ g [5,6] at temperature levels from 5 to 300 K and ambient pressures of 10^−10^ to 10^−5^ Torr.

Selected excitation and measurement methods are described below as examples. In the inductive method, a nano-resonator is placed in a magnetic field and a broadband alternating current is passed through the resonator so that the Lorentz force excites oscillation. The oscillation, in turn, induces a voltage that is amplified and measured to record the oscillation. A disadvantage, however, is the superposition of the measurement signal and the excitation. With the aid of special circuit variants, the feedback effect of the exciting signal on the measurement signal can be reduced [7,8].

**Table 1:** Selected publications on resonant nano-mass detection systems.

	Material	Beam Support	Resolution [g]	ω_0_ [MHz]	Co-Resonant	Excitation	Measurement Principle
[9]	Silicon	single-sided	5.5 × 10^−15^	1...10	-	photothermal	interferometer
[10]	Silicon	double-sided	n.a.	1000	-	capacitive	capacitive
[11]	SiC	double-sided	2.53 × 10^−18^	32.8	-	capacitive	capacitive
[12]	CNT	single-sided	1.3 × 10^−22^	328.5	-	capacitive	capacitive
[13]	CNT	double-sided	2.5 × 10^−20^	125	-	capacitive	reflection
[14]	SiC	double-sided	n.a.	428	-	capacitive	capacitive
[5]	CNT	double-sided	1.7 × 10^−24^	2000	-	capacitive	reflection
[6]	CNT	single-sided	1.7 × 10^−24^	12 × 10^4^	-	capacitive	reflection
[15]	Graphene	double-sided	1.41 × 10^−21^	1.1	+	piezoelectric	piezoresistive
[16]	Silicon	double-sided	1.7 × 10^−21^	20...120	+	capacitive	piezoresistive
[17]	Nano-crystalline	single-sided	10^−18^	12 × 10^3^	+	capacitive	reflection
[18]	Silicon	single-sided	10^−12^	1.1	+	piezoelectric	piezoelectric

In the electrostatic method, a nano-resonator is excited by an electrostatic field. A voltage between the nanostructure and electrodes, which are arranged in parallel, usually generates the field. In the case, either a broadband source or a source with an adjustable frequency can serve as the voltage source, whereby the frequency must be monitored for the latter, e.g., via a phase-locked loop. The resonant frequency is detected by measuring the change in capacitance between the nano-resonator and the counter electrode. The capacitance change can be measured by measuring the frequency of the displacement current at the capacitor with a transimpedance amplifier [4,7,19]. Self-oscillating active electrical circuits that integrate the nano-resonator circuitry are also used.

Another method for determining the resonant frequency is based on the reflection measurement at an integrated transistor, which consists of a semiconducting nano-resonator that is mechanically clamped and electrically contacted on both sides with a counter electrode as gate. The reflection of an alternating signal at the electrical RLC resonant circuit with the integrated nano-transducer is then measured. Particle adsorption at the nano-resonator leads to a change in resistance and capacitance at a constant voltage between the gate and the oscillator and thus to a change in the reflection. However, this method requires a complex design of the nano-resonator as a transistor [20,21].

Other methods for measuring oscillations are based on optical interferometers in which an incident light beam is reflected by the nano-oscillator and superimposed with a reference beam. Based on the time-varying interference pattern, the vibration can be analyzed. The excitation of the nano-resonator can also be done photothermally with a laser by heating and expanding the nanostructure [9,22].

Piezoelectric methods, e.g., see [18], are based on the mechanical excitation of the nano-resonator via the deformation of piezoelectric nanostructures when a voltage is applied. Similar to electrostatic methods, the electrical voltage for vibration excitation can be generated via a self-oscillating active electrical circuit, e.g., see [23]. The piezoelectric approach of vibration excitation and measuring the resonant frequency was applied in this study.

A fundamental remaining challenge is designing and, in particular, fabricating a highly accurate and reproducible mass detection systems that would allow for the detection of the smallest masses in a simple way with minimal effort, so that, for example, large arrays or single sensors for process or environmental monitoring become possible.

**Figure 1:**
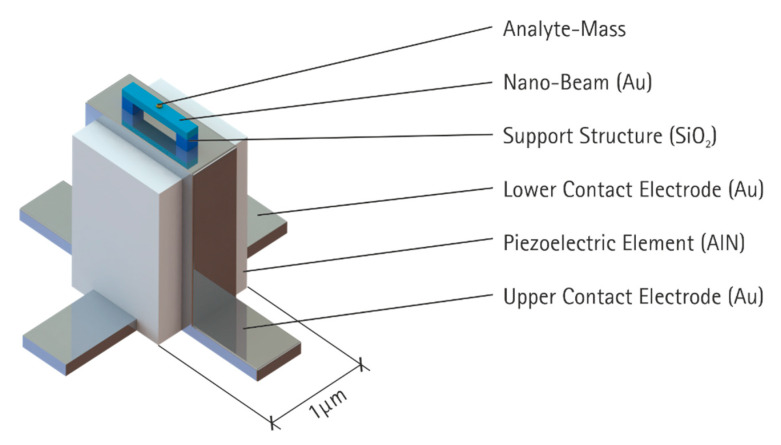
Novel setup for a piezoelectric co-resonant mass-detector. The piezoelectric element was designed as a longitudinal resonator, and the nano-beam was operated in a bending mode.

In this preliminary concept study, we focused on co-resonance systems, utilizing the advantage that the frequency detection can be relocated from the tiny nano-resonator to a second somewhat-large resonator, which is the host platform for the nano-resonator. This concept is extended using a piezoelectric element for the larger resonator. If well-designed, this would allow for the use of the piezoelectric effect for both the actuation of the nano-beam and the detection of its resonance frequency. The fabrication of nano-beams that are attached to the tips of larger beams—which is the usual setup of co-resonant systems—is rather complicated. We therefore propose a different approach. Here, the larger resonator was designed in a longitudinal fashion. In addition to the ease of fabrication, this leads to an even vibration distribution. On a respective surface, the sensitivity to additional masses is approximately equal across the entire surface. Since the surface is the free end, the vibration amplitude is the maximum (anti-nodal-plane) of the longitudinal vibration. This gives a certain degree of freedom for the setup of the nano-resonator. A schematic sketch of the novel concept is given in Figure 1.

In this study, we investigated the feasibility of this novel concept. This included studying relevant design parameters. For this purpose, three model approaches are presented in the Materials and Method section and discussed in the Results section. The goal was to reveal parameter ranges that allow for a practical realization of the proposed concept. Therefore, the sensitivity of the vibration modes, their coupling, dependence on mass, and stiffness properties were investigated by utilizing an equivalent model. The piezoelectric effect also needed to be considered in order to evaluate the sensing performance. Using a finite element (FE) model, the geometry of the proposed structure was designed and specified. The resulting model-based findings were used to evaluate the characteristic of the novel setup.

## 2. Materials and Methods

The first step was the selection of useful eigenmodes. For this purpose, we investigated their frequency sensitivity of the bending and longitudinal modes. Those studies used a point mass representing the analyte mass, located in the anti-node of the vibration. Furthermore, an equivalent model for the investigation of co-resonant vibrations was developed. This was used to compute the major parameter affecting the vibration. Finally, the parameter of the later finite element model was presented, and this model was then utilized to show the feasibility of the novel concept.

### 2.1. Sensitivity of Relevant Mode Shapes

To investigate the influence of the analyte mass on the resonance frequency for the interesting mode shapes, we used the well-known, analytically-derived formulas given in many text-books; here, we used the reference book [24]. For the beam, three configurations were investigated: clamped-free (cf), with the extra mass at the free end, and clamped–clamped (cc) and pinned–pinned (pp), both with the extra mass at the center position. The resonance frequencies f, where the index Bxx indicates the bending vibration and the respective boundary conditions, are as follows.

(1) $$f_{\mathrm{Bcf}}=\frac{1}{2\;\pi }\sqrt{\frac{3EI}{l^{3}\left(m_{n}+0.24\;m\right)}},$$

(2) $$f_{\mathrm{Bcc}}=\frac{4}{\pi }\sqrt{\frac{3EI}{l^{3}\left(m_{n}+0.37\;m\right)}},$$

(3) $$f_{\mathrm{Bpp}}=\frac{4}{\pi }\sqrt{\frac{3EI}{l^{3}\left(m_{n}+0.49\;m\right)}},$$

where E is the modulus of elasticity, l is the free length of the beam, I is the area moment of inertia, m is the mass of the uniform beam, and $m_{n}$ is the analyte mass. In addition to the bending, we also considered the longitudinal vibration. The boundary conditions of interest were: clamped-free (cf) and free–free (ff), both with an extra mass at one free side. The resonance frequencies were mathematically determined as follows:

(4) $$f_{\mathrm{Lcf}}=\frac{\lambda_{\mathrm{cf}}}{2\;\pi \;l}\sqrt{\frac{E}{\varrho }}\;\mathrm{with}\;c\mathrm{ot}\left(\lambda_{\mathrm{cf}}\right)=\frac{m_{n}}{m}\lambda_{\mathrm{cf}},$$

(5) $$f_{\mathrm{Lff}}=\frac{\lambda_{\mathrm{ff}}}{2\;\pi \;l}\sqrt{\frac{E}{\varrho }}\;\mathrm{with}\;\tan\left(\lambda_{\mathrm{ff}}\right)=-\frac{m_{n}}{m}\lambda_{\mathrm{ff}},$$

where ϱ is the density and $\lambda_{\mathrm{cf}}$ and $\lambda_{\mathrm{ff}}$ are the dimensionless frequency parameters that can be found by solving the given transcendental equation, where we are interested in the first solution for the fundamental eigenfrequency. In case of $m_{n}=0$, the parameters are: $\lambda_{\mathrm{cf}}=\pi /2$ and $\lambda_{\mathrm{ff}}=\pi$.

For the investigation of the frequency shift dependent on the analyte mass, we computed the ratio of the loaded and unloaded resonance frequencies (marked with the index 0) and subtracted 1 to obtain the relative frequency change. Additionally, we introduced the mass ratio $\gamma =m_{n}/m$ and obtained the relative frequency shift β for the five cases:

(6) $$\beta_{\mathrm{Bcf}}=\frac{f_{\mathrm{Bcf}}}{f_{\mathrm{Bcf},0}}-1=\sqrt{\frac{1}{\frac{\gamma }{0.24}+1}}-1$$

(7) $$\beta_{\mathrm{Bcc}}=\frac{f_{\mathrm{Bcc}}}{f_{\mathrm{Bcc},0}}-1=\sqrt{\frac{1}{\frac{\gamma }{0.37}+1}}-1$$

(8) $$\beta_{\mathrm{Bcf}}=\frac{f_{\mathrm{Bcf}}}{f_{\mathrm{Bcf},0}}-1=\sqrt{\frac{1}{\frac{\gamma }{0.49}+1}}-1$$

(9) $$\beta_{\mathrm{Lcf}}=\frac{f_{\mathrm{Lcf}}}{f_{\mathrm{Lcf},0}}-1=\frac{2\;\lambda_{\mathrm{cf}}}{\pi }-1\;\mathrm{with}\;c\mathrm{ot}\left(\lambda_{\mathrm{cf}}\right)=\gamma \lambda_{\mathrm{cf}},$$

(10) $$\beta_{\mathrm{Lff}}=\frac{f_{\mathrm{Lff}}}{f_{\mathrm{Lff},0}}-1=\frac{\lambda_{\mathrm{ff}}}{\pi }-1\;\mathrm{with}\;\tan\left(\lambda_{\mathrm{ff}}\right)=-\gamma \lambda_{\mathrm{ff}},$$

**Figure 2:**
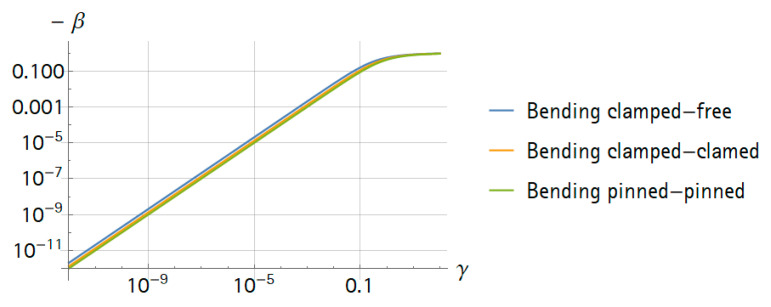
Relative frequency changes (−β) of bending modes over mass ratio (γ).

In the special case that the mass ratios were much smaller than one, all frequency ratios were very similar and β was in the same order of magnitude as the mass ratio. The results for the bending frequencies are summarized in Figure 2. For small mass ratios, the function was linear, and for larger mass ratios (≈γ>0.05), the approximation of the point mass was insufficient.

As an example, the following approximation could be made using the formulas. If an analyte mass is a billion-times smaller than the mass of a resonator, the frequency resolution of the measurement electronics has to be better than a billionth of the resonance frequency.

Thus, the mass of the nano-resonator is crucial for the mass resolution, especially when considering the required frequency resolution of the measurement electronics. If, at a resonance frequency of 1 GHz, 1 mHz (the resolution of a good impedance analyzer) is detectable, the detection of a mass 10^12^ times smaller than the resonator is theoretically possible. In other words, to detect a yoctogram, the mass of the nano-resonator must be smaller than a picogram. However, many disturbing influences (temperature, pressure, etc.) can negatively affect the resolution.

### 2.2. Equivalent Model for A Piezoelectric Co-Resonant Vibration System

The direct excitation and measurement of a nano-resonator is challenging; therefore, we used and extended the co-resonance concept. The main idea was to couple a smaller and a larger resonator in such a way that a change of the vibration properties of the smaller resonator could be measured by the vibration properties of the larger resonator. For this purpose, two vibrational structures were selected (typically two bending beams) and independently tuned to the same resonance frequency. Then, the smaller beam was placed on the larger beam at a position where the selected eigenmode of the larger beam had the highest amplitude (anti-node). At this position, the impact of the smaller beam on the larger beam was maximized. Due to the physical combination of the two structures, a new system was created with new properties, with respect to eigenfrequencies and mode shapes.

**Figure 3:**
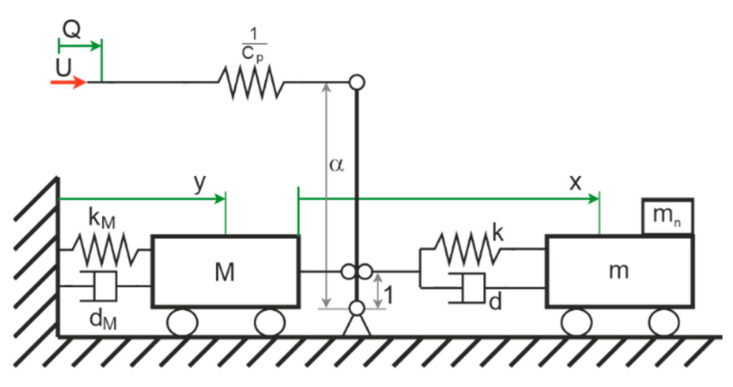
Simplified equivalent model for the co-resonant mass detector.

For analytical purposes, we applied a simplified model based on a system with ideal lumped parameters; two degrees of freedom; and discrete stiffnesses, damper, and masses. The piezoelectric effect—which is used for actuation and sensing—can be included through the second electro-mechanical analogy, e.g., see [25]. By utilizing a parameter comparison of the equations for electrical and mechanical discrete components, the analogy was traditionally derived. In the herein-used second analogy, displacement is equivalent to electric charge and force is equivalent to current. An ideal transmission element such as a lever can transfer energy from one domain to the other. However, the lever parameter consequently has a unit. The model is depicted in Figure 3.

The model has three coordinates: the absolute position y of the lager mass M, the position x of the smaller mass m with respect to y, and the electric charge Q. The larger mass M is inertially connected though the stiffness $k_{M}$ and the damper $d_{M}$. The stiffness k and the damper d connects M and m. The analyte mass is $m_{n}$ adding to m. The lever with the unitized factor α couples the mechanic and the electric domain. The capacity of the piezoelectric element is *C*_p_, and the system is driven by the voltage *U*. Utilizing complex amplitudes x(t)=Re(x^ejφejΩt)=Re(x^_ejΩt) and the imaginary unit j and *Ω* as angular excitation frequency, for the equations of motion follows for the harmonic excitation:

(11) $$\left(-\left(m+m_{n}\right)\Omega^{2}+j\Omega d+k\right)\underline{\hat{x}}=\left(m+m_{n}\right)\Omega^{2}\underline{\hat{y}},\;\left(-M\Omega^{2}+j\Omega d_{M}+k_{M}\right)\underline{\hat{y}}-\left(j\Omega d+k\right)\underline{\hat{x}}=\alpha \underline{\hat{U}},\;\mathrm{and}\;\frac{1}{C_{p}}\underline{\hat{Q}}-\frac{\alpha }{C_{p}}\underline{\hat{y}}=\underline{\hat{U}}.$$

Additionally, we defined the two decoupled eigenfrequencies with $m_{n}=0$:

(12) $$\omega_{1}=\sqrt{\frac{k}{m}}$$

(13) $$\omega_{2}=\sqrt{\frac{k_{M}}{M}}$$

For further analysis, transfer-functions were also needed; here, we used the current amplitude $\underline{\hat{i}}=j\Omega \underline{\hat{Q}}$:

(14) $$G_{\mathrm{iU}}=\frac{\underline{\hat{i}}}{\underline{\hat{U}}}=\frac{j\;\Omega {\;\alpha }^{2}\left(-m\Omega^{2}+j\Omega d+k\;\right)}{kk_{M}+j\left(d_{M}k+dk_{M}\right)\Omega -\left(dd_{M}+\left(k+k_{M}\right)m+k_{M}\right)\Omega^{2}-j(d_{M}m+\;d\left(m+M\right))\Omega^{3}+mM\Omega^{4}\;}+j\Omega C_{p}$$

(15) $$G_{\mathrm{yU}}=\frac{\underline{\hat{y}}}{\underline{\hat{U}}}=\frac{\alpha \left(-m\Omega^{2}+j\Omega d+k\;\right)}{kk_{M}+j\left(d_{M}k+dk_{M}\right)\Omega -\left(dd_{M}+\left(k+k_{M}\right)m+k_{M}\right)\Omega^{2}-j(d_{M}m+\;d\left(m+M\right))\Omega^{3}+mM\Omega^{4}\;}$$

(16) $$G_{\mathrm{xU}}=\frac{\underline{\hat{x}}}{\underline{\hat{U}}}=\frac{\alpha m\Omega^{2}}{kk_{M}+j\left(d_{M}k+dk_{M}\right)\Omega -\left(dd_{M}+\left(k+k_{M}\right)m+k_{M}\right)\Omega^{2}-j(d_{M}m+\;d\left(m+M\right))\Omega^{3}+mM\Omega^{4})\;}$$

### 2.3. Finite Element Simulation

To confirm that the novel idea of combining a longitudinal resonator with a bending resonator was promising approach for future nano-mass detectors, a 3D-FE model was built in ANSYS for first investigations. In this step, the substrate was included as an elastic foundation. The material parameters used for aluminum nitride (including the piezoelectricity) and silicone dioxide stemmed from [26] and are given in Table 2. For the analysis, we used modal and harmonic simulations.

## 3. Results and Discussion

This section is divided in two subsections. First, the equivalent model is used to investigate the parameter impacts on the effective coupling between the two resonators. These results are then discussed in conjunction with theoretical sensitivity. Second, the feasibility of the concept, as presented in Figure 1, is studied.

### 3.1. Evaluation of Equivalent Model

For the investigation of the equivalent model, we concentrated on the interaction of the two modes, the frequency responses, and the sensitivity to the analyte mass.

#### 3.1.1. Coupling of the Co-Resonant Masses

It is clear that $\omega_{1}=\omega_{2}$ leads to the best interaction between the modes; this is well-known for a mechanically-tuned mass damper. For our purposes, the coupling had to be sufficiently large so that a resonance frequency shift of the nano-resonator changed the resonance frequency of the larger resonator.

In our case, the mass M was always larger than *m*, and the coupling decreased with a smaller mass ratio κ=m/M. This was clear for a very large M, where any motion of *m* was negligible for the behavior of M. In the case that *m* and M were similar in weight, an interaction existed. One possibility to show this interaction was to investigate the system’s eigenfrequencies.

For this, we focused on the undamped system. The mass ratio κ=m/M was used to scale the stiffness $k_{M}$ to ensure that both uncoupled eigenfrequencies remained equal $\omega_{1}=\omega_{2}$ in the first place. For the investigation of a mistuning of the two eigenfrequencies, the factor Λ was introduced so that $\omega \prime_{2}=\Lambda \omega_{1}$. For simplicity, we used the coordinate $x_{A}$ for the mass m with respect to the reference frame. With Q≠0 and U=0 as the boundary condition for the electric port and $m_{n}=0$ the mass and stiffness matrix, the following equation applies:

(17) $$M=\left(\begin{array}{cc} m & 0\\ 0 & m/\kappa \end{array}\right),\;\;K=\left(\begin{array}{cc} k & -k\\ -k & \Lambda^{2}\;k/\kappa \end{array}\right),\;x=\left(\begin{array}{c} x_{A}\\ y \end{array}\right).$$

The coupled system has the two eigenfrequencies with $\omega_{1}^{2}=\frac{k}{m}$:

(18) $$\omega_{L}^{2}=\frac{1}{2}\omega_{1}^{2}\;\left(1+\Lambda^{2}-\sqrt{4\kappa +{\left(\Lambda^{2}-1\right)}^{2}}\right)$$

(19) $$\omega_{H}^{2}=\frac{1}{2}\omega_{1}^{2}\;\left(1+\Lambda^{2}+\sqrt[{}]{4\kappa +{\left(\Lambda^{2}-1\right)}^{2}}\right)$$

**Figure 4:**
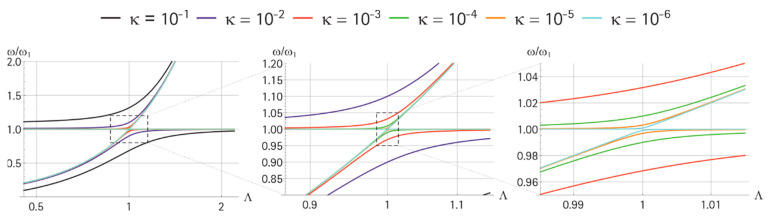
Parameter dependence of the system eigenfrequencies $\omega_{L}$ and $\omega_{H}$ for a variation of the frequency-mistuning parameter Λ. The zoom-level is increased from the left to the right in the plot. The used parameters were: m = 1 kg, k = 1 N/m, and $\kappa =10^{-6}\ldots 10^{-1}$.

The results are given in Figure 4. As expected, for very small κ, the lower (L) and higher (H) system eigenfrequencies ($\omega_{L}$ and $\omega_{H}$, respectively) crossed when Λ=1. For this κ, both frequencies behaved as uncoupled. The larger κ got, the more the coupling affected the results, and only for large mismatching, the resulting frequencies were close to those of the uncoupled case.

Interestingly, the more we zoomed in (figures from left to right), especially for smaller κ, a coupled region appeared in approximately 0.99<Λ>1.01, which showed that, even then, the co-resonance concept might be useful. However, the matching required extreme precision: for $\kappa =10^{-4}$, $\omega_{1}\;$and $\omega_{2}$ should have deviated less than ±1%. Consequently, the larger resonator should be as small as possible.

#### 3.1.2. Frequency Response

**Figure 5:**
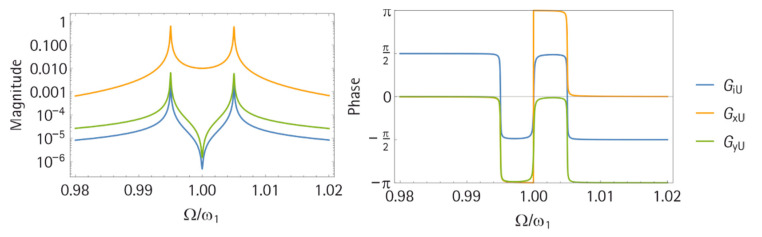
Frequency response for $G_{\mathrm{iU}}$, $G_{\mathrm{yU}}$, and $G_{\mathrm{xU}}$ in magnitude (left) and phase (right). The used parameters were m = 1 kg, m_n_ = 0, k = 10 N/m, M = 10^4^ kg, k_M_ = 10^5^ N/m, D = d_M_ = 5 × 10^−4^ Ns/m, C_p_ = 10 nF, and α = 0.1 N/V.

The harmonic frequency response was easily computed with Equations (14)–(16). Figure 5 shows an exemplary result. This case was for $\kappa =10^{-4}$, where the two system resonances $\omega_{L}$ and $\omega_{H}$ were clearly visible in all three curves, and in $G_{\mathrm{iU}}$ and $G_{\mathrm{yU}}$, an additional anti-resonance $\omega_{A}$ appeared at $\omega_{1}$, which is the optimal vibration migration frequency for the application of a tuned mass damper. Remarkably, there was no further antiresonance frequency in $G_{\mathrm{iU}}$ that could be expected, possibly due to the already high transmission factor α=0.1. This is promising because the corresponding phase zero crossing can potentially be used to track $\omega_{A}$ with a phase feedback control. This is also the case for the two phase zero crossings at $\omega_{L}$ and $\omega_{H}$. For these two frequencies, self-oscillation circuits can be also applied to operate the system at resonance.

**Figure 6:**
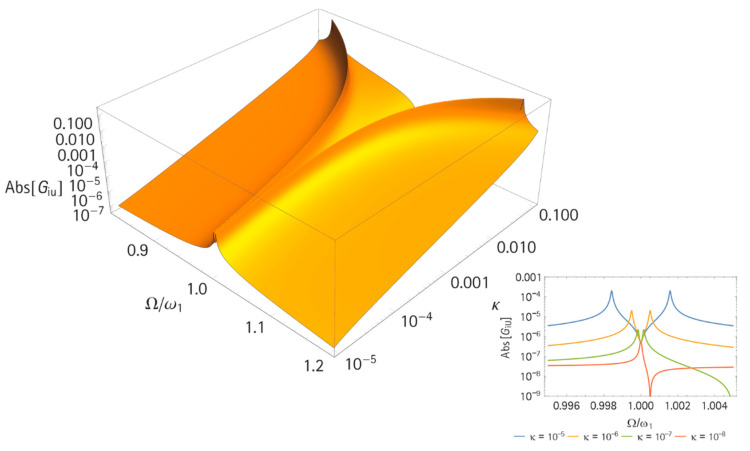
Magnitude of the admittance ($G_{\mathrm{iU}}$) for varying κ values, (left) 3D representation for a large range of κ, (right) 2D plot for very small mass ratios, and $\kappa =10^{-8}\ldots 10^{-5}$. The used parameters were m = 1 kg; m_n_ = 0; k = 10 N/m; M = m/κ; k_M_ = k/κ; D = d_M_ = 5 × 10^−4^ Ns/m; C_p_ = 10 nF; and α = 0.1 N/V.

A closer look at the impact of the mass ratio κ in Figure 6 shows that the difference between $\omega_{L}$ and $\omega_{H}$ decreased for decreasing κ values. The main issue with a small frequency difference is that the coupling range becomes smaller and the system is more affected by production tolerances. Furthermore, the magnitude of $G_{\mathrm{iU}}$ increased with decreasing κ values, which led to an elimination of the phase zero crossing in $G_{\mathrm{iU}}$ at $\omega_{A}$. If κ. became smaller than $10^{-8}$, the two peaks of $\omega_{L}$ and $\omega_{H}$ joined, and both resonators decoupled. This is shown in Figure 6 on the right side. For a κ value so small, the frequency response resembled the response of a single piezoelectric element. However, the result was very promising for the co-resonance concept, since even for extremely small mass ratios κ (but still above $\kappa =10^{-7}$), coupling is possible. Nevertheless, for smaller κ values, the initial tuning of $\omega_{1}\;$and $\omega_{2}$ must be more accurate.

**Figure 7:**
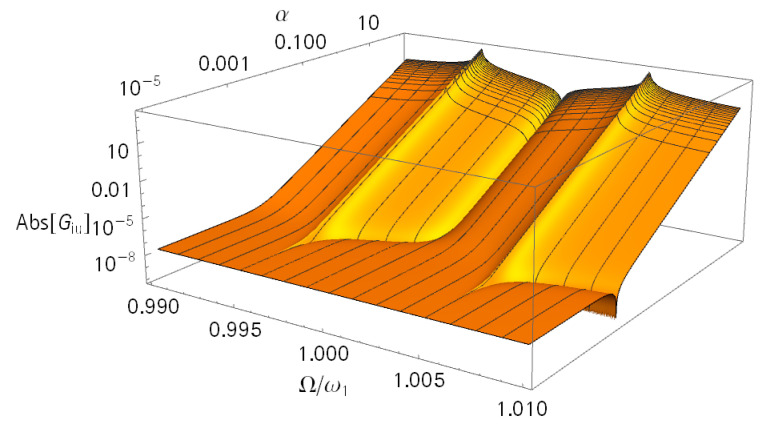
Magnitude of the admittance ($G_{\mathrm{iU}}$) for varying α values. The used parameters were m = 1 kg; m_n_ = 0; k = 10 N/m; M = m/κ; k_M_ = k/κ; κ = 10^−4^; D = d_M_ = 5 × 10^−4^ Ns/m; C_p_ = 10 nF; and α = 0.1 N/V.

It is desirable for the operation of a nano-mass detector to have a phase zero crossing of $G_{\mathrm{iU}}$ at $\omega_{A}$. The transmission factor α was found being the main parameter in this study. Figure 7 shows its impact on the magnitude of $G_{\mathrm{iU}}$ for a constant $\kappa =10^{-4}$. For a large α. value, the antiresonance was clearly at $\omega_{1}$; with a decreasing value of α, the $\omega_{A}$ moved toward $\omega_{L}$. The piezoelectric coupling was not sufficient anymore to track the mechanical antiresonance. The transmission factor, however, was not the only reason for the shifting $\omega_{A}$. When the term $j\Omega C_{p}$ in Equation (14) became dominant, the other summand with the quadratic factor α significantly decreased. Eventually, $j\Omega C_{p}$ limited this decrease. For our application, we consequently looked for a large α while keeping $C_{p}$ within limits.

#### 3.1.3. Sensitivity on Analyte Mass

**Figure 8:**
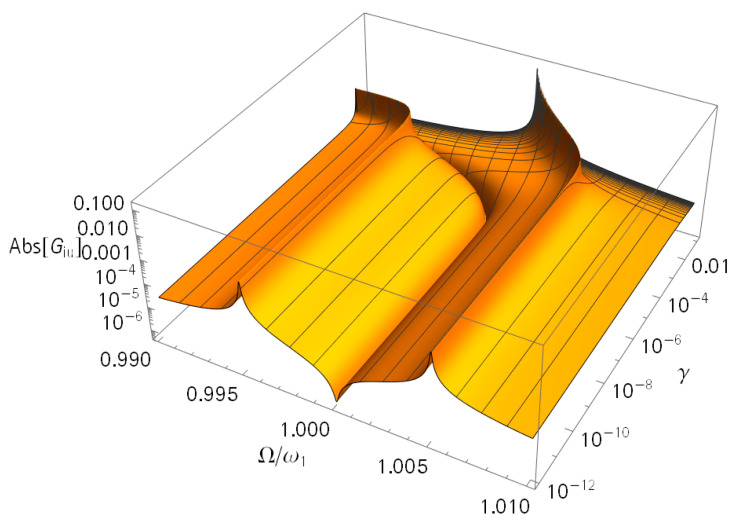
Magnitude of the admittance ($G_{\mathrm{iU}}$) for varying γ values. The used parameters were m = 1 kg; m_n_ = γm; k = 10 N/m; M = m/κ; k_M_ = k/κ; κ = 10^−4^; D = d_M_ = 5 × 10^−4^ Ns/m; *C*_p_ = 10 nF; and α = 0.1 N/V.

The most important factor of a system is the frequency sensitivity to the analyte mass. First, the corresponding frequency response can be observed in Figure 8. Here, γ denotes $m_{n}/m$ and an increasing γ corresponds to an increasing analyte mass. There was no obvious change in the response for small γ. This was in agreement with the findings in Figure 2.

**Figure 9:**
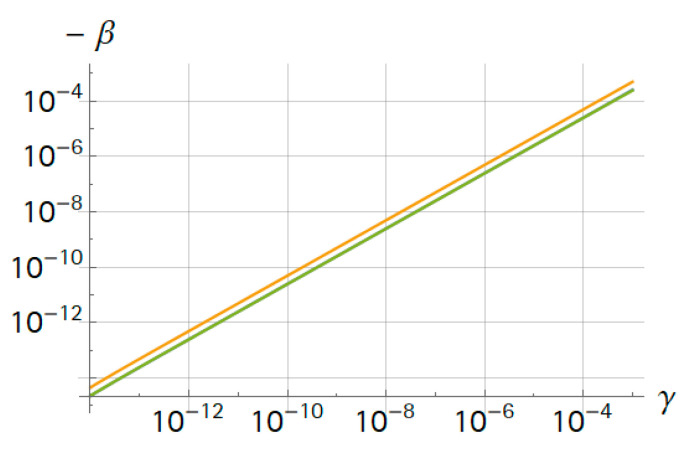
Relative frequency changes (−β) due to the mass ratio (γ) for the equivalent model. The used parameters were m = 1 kg; m_n_= γ m; k = 10 N/m; M = m/κ; k_M_ = k/κ; κ = 10^−4^; D = d_M_ = 5 × 10^−4^ Ns/m; *C*_p_ = 10 nF; and α = 0.1 N/V.

Second, in order to visualize the frequency shift, we tracked three frequencies—both resonance frequencies and the antiresonance. Mass detection is based on finding the zero crossings in the phase of $G_{\mathrm{iU}}$. These frequencies could be tracked by a PLL (phase-locked-loop) control in a future device. With this data, we computed the relative frequency shift β again (Figure 9). Interestingly, the shift of the antiresonance frequency was twice as large as the shift of the resonance frequencies, where κ was again $10^{-4}$. Computations showed that this was independent of κ in the range of $10^{-5}<\kappa <10^{-2}$ (data not shown). Compared to the results in Figure 2, the sensitivity was about one order of magnitude lower due to the co-resonance structure, but detection and sensing were easier compared to a sole nano-resonator. Moreover, the piezoelectric approach further simplified the system. In the future, the dimensions of the nano-resonator could be further reduced. This is not possible for a sole nano-resonator due to issues related to the actuation and measurement of the vibration. Therefore, for co-resonant systems, a higher sensitivity seems to be within reach.

### 3.2. Device Design

With the knowledge from the equivalent model, an important step towards a specific geometry could now be made. Here, we also considered the fabrication process to ensure that the design is suited for efficient batch production. In contrast to previous investigations on co-resonant systems, we propose a combination of a longitudinal and a bending resonator. This concept offers several advantages. In particular, the fabrication of the electrodes for the piezoelectric longitudinal transducer is less complicated. However, there is a drawback: the vibration coupling of the longitudinal resonator to the substrate has to be considered in the design process. Our fundamental setup is shown in Figure 1. We selected AlN as a piezoelectric material due to its good acoustic properties and its well-established fabrication process. The nano-beam was made of gold (Au) and was supported by two silicon dioxide elements. The piezoelectric element was 1 × 1 × 1.4 µm in size. The Au bottom electrode was buried by the AlN. The top electrode was arranged perpendicular to the bottom electrode and covered the top and the two sides of the AlN element. The nano-beam had a cross-section of 320 × 210 nm and a free length of 250 nm. The SiO_2_ support elements had a height of 300 nm and a footprint of 750 × 200 nm.

**Figure 10:**
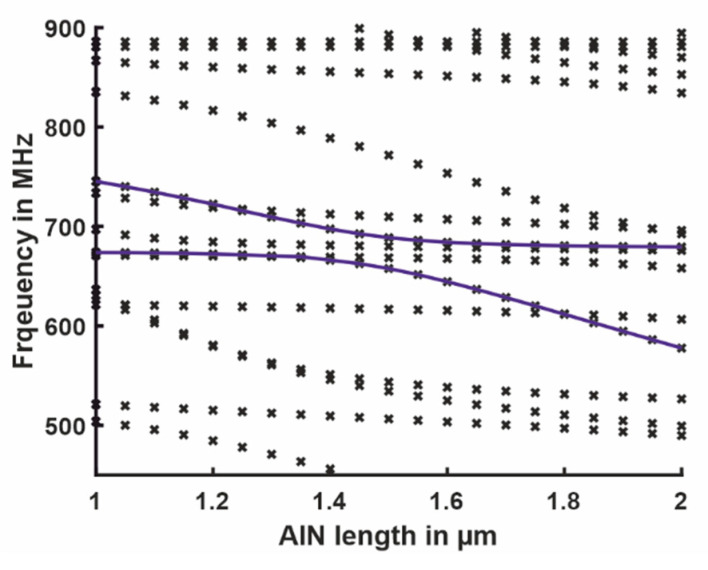
Parameter dependence of all eigenfrequencies on the different length of the piezoelectric element in the frequency range from 450 to 900 MHz, as indicated by makers. The lines show the change of the two utilized eigenmodes $\omega_{L}$ and $\omega_{H}$.

The whole system was modeled in ANSYS Workbench, where the piezoelectric effect was considered by using the element solid226. The mode shapes were computed using modal analysis. An independent design of the resonator dimensions could not be done since the effective mass of both resonators should not have extremely differed. Furthermore, the boundary conditions of the nano-beam significantly depended on the elastic properties of the longitudinal resonator. For the design of the longitudinal resonator, all relevant components needed to be considered in the model, including the ground plane and the supporting elements of the nano-beam. Consequently, the components had to be simultaneously designed to obtain a suited setup. The selected dimensions were a result of an empirical parameter study. For example, Figure 10 depicts the resonance frequencies as a function of the length (direction of vibration) of the piezoelectric AlN longitudinal resonator. Each marker indicates an eigenfrequency of the system. The result of the modal analysis of the 3D FE Model did included not only the two coupled eigenmodes *ω*_L_ and *ω*_H_ but also all other modes in the investigated frequency range. Due to the geometry change, the order of the eigenmodes varied. The coupled modes were identified in the results and are connected by the blue solid lines. Again, the mode coupling was clearly visible, especially when compared to Figure 4. To check if the other modes (markers without line) in the interesting frequency range were also interacting with the two coupled eigenmodes, harmonic simulations (where the piezo was driven electrically) will be carried out next to the modal analysis. Those results in Figure 12 show that only the two coupled longitudinal-bending modes were excitable. In the same way, additional geometrical parameters were simulated. To obtain the best configuration, a multi parameter optimization is needed in the future.

**Figure 11:**
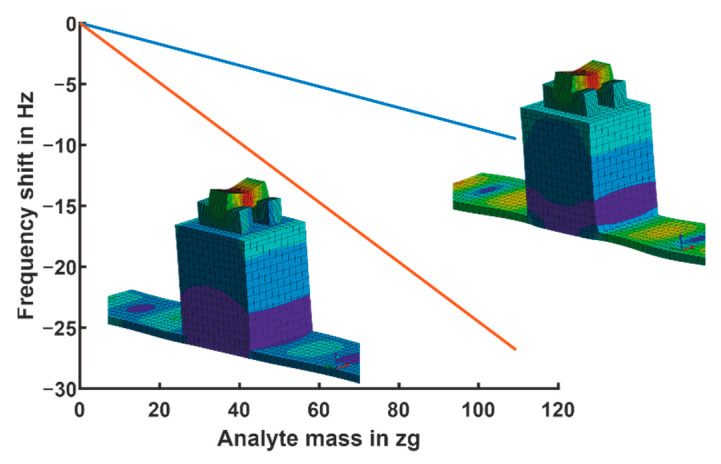
Absolute frequency change of $\left(\omega_{L}-\omega_{L,0}\right)/\left(2\pi \right)$ and $\left(\omega_{H}-\omega_{H,0}\right)/\left(2\pi \right)$ due to an analyte mass attached to the top center of the nano-beam and corresponding coupled eigenmodes.

The frequency shift, due to the analyte mass, of the two coupled modes have been computed by modal analysis. The results are shown in Figure 11 with unloaded frequencies of 666 and 697 MHz. The frequency shift is determined by $\left(\omega_{L}-\omega_{L,0}\right)/\left(2\pi \right)$ and $\left(\omega_{H}-\omega_{H,0}\right)/\left(2\pi \right)$, respectively, where the index “0” indicates the unloaded case. The uncoupled frequencies could not be determined since the boundary conditions were too complex. To estimate the sensitivity to the analyte mass, a small element (25 × 25 × 25 nm) was attached to the top center of the nano-beam. The weight was adapted by changing its density.

The sensitivity was approximately 250 Hz/ag. Due to the more complex geometry compared to the equivalent model, the sensitivity of both modes was different. Consequently, we expected a detectable mass of 400 yg ($4\times 10^{-22}$ g) if the measurement electronics had a 100 mHz resolution. According to Table 1, this was in the range of the carbon-nanotube based detectors, especially the detector of [12] showing an approximately three times better resolution. The advantage of the novel design is its simplicity in setup, that allows for a rather easy fabrication compared to the already known devices. For the detection of a single digit yoctogram, we aim for both further miniaturization and an improvement in the frequency resolution of the measurement electronics towards a few mHz. The hitherto theoretical concept therefore has the potential to achieve a resolution in the range of the atomic mass unit ($1.66\times 10^{-24}$ g) if both measures can be successfully implemented.

However, according to the 3σ definition of the limit of detection (LOD), detecting $1.66\times 10^{-24}$. g does not only require a frequency resolution of the measurement electronics of a few mHz for improved sensitivity but also a frequency noise of the zero signal of $\sigma =1.66\times 10^{-24}\times 1000\times 10^{18}\frac{\mathrm{Hz}}{g}=1.66\mathrm{mHz}$ at a given sensitivity, e.g., of 1000 $\frac{\mathrm{Hz}}{\mathrm{ag}}$, which seems quite challenging.

**Figure 12:**
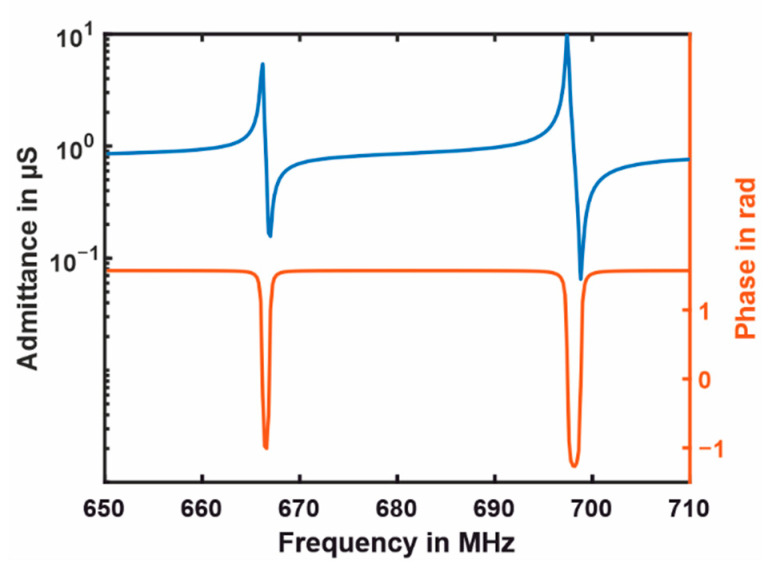
Current amplitude in the magnitude and phase of the co-resonant vibration system.

Electrical behavior is crucial for the sensing process; therefore, a harmonic simulation was computed. The system was driven with a 1 V amplitude, and the corresponding frequency response $G_{\mathrm{iU}}$ is shown in Figure 12. It was evident that the capacity dominated (compare Figure 7), hence two clear resonance–anti-resonance combinations appeared. Both had a phase zero crossing, and the minimum phase was about −60°, which was sufficient for both PLL tracking and a self-oscillation approach. The dominant capacity did not allow for the tracing of the $\omega_{1}=\omega_{A}$ frequency by a PLL circuitry for this parameter combination. However, the simulated results underlined that the novel concept of combining a longitudinal and a bending resonator is feasible and very promising.

## 4. Conclusions

With the presented theoretical investigation, the feasibility of the novel combination of longitudinal and bending modes for co-resonant nano-mass detectors has been demonstrated. The new design simplifies significantly fabrication compared to many state-of-the-art structures used in other approaches. The sensitivity was, at first sight, about just one order of magnitude smaller that in non-co-resonant systems. Nevertheless, due to the simple design of the nano-beam, it has potential for its size to be further reduced. The direct application of the piezoelectric element for actuation and sensing appears very promising and also simplifies the system. The detection of the mechanical anti-resonance in $G_{\mathrm{yU}}$ through the phase zero crossing of $G_{\mathrm{iU}}$ seems to be possible but very challenging. This has the potential of an additional factor of 2 in the resolution.

These results motivate the next steps: to prove the model-based findings in the laboratory. Here, we will work on the presented device with an expected resolution of 400 yg. For this, the design will be further improved hand-in-hand with the technology development of the fabrication process. The novel device will then be experimentally characterized and evaluated.

## Figures and Tables

**Table 2:** Used material parameters. AlN: aluminum nitride, Au: gold, SiO_2_: silicon dioxide.

Property	Unit	AlN	Au	SiO_2_
Density	kg/m^3^	3512	19320	2200
Poisson ratio	-	0.3	0.42	0.17
Stiffness coefficient c_11_	GPa	345		
Stiffness coefficient c_12_	GPa	125		
Stiffness coefficient c_13_	GPa	120		
Stiffness coefficient c_33_ /Elastic modulus	GPa	395	79	70
Stiffness coefficient c_44_	GPa	118		
Stiffness coefficient c_66_	GPa	110		
Piezoelectric constant e_31_	C/m^2^	−0.58		
Piezoelectric constant e_33_	C/m^2^	1.55		
Piezoelectric constant e_15_	C/m^2^	−0.48		
Relative permittivity ε/ε_0_	-	11		

## Data Availability

The data are available upon request.
